# The Dual Role of NOX4 in Cardiovascular Diseases: Driver of Oxidative Stress and Mediator of Adaptive Remodeling

**DOI:** 10.3390/antiox14091137

**Published:** 2025-09-19

**Authors:** Pauline Labbé, Eric Thorin, Nathalie Thorin-Trescases

**Affiliations:** 1 Research Center Montreal Heart Institute, Université de Montréal, Montréal, QC H1T 1C8, Canada; eric.thorin@umontreal.ca (E.T.); nathalie.trescases@gmail.com (N.T.-T.); 2 Centre Hospitalier Universitaire Sainte-Justine, Université de Montréal, Montréal, QC H3T 1C5, Canada; 3 Department of Surgery, Université de Montréal, Montréal, QC H3C 3J7, Canada

**Keywords:** NOX4, heart diseases, oxidative stress, redox sensor, therapeutic target

## Abstract

NADPH oxidase 4 (NOX4) plays a crucial role in regulating cardiac function and pathology through its involvement in oxidative stress, fibrosis, and maladaptive remodeling. Studies have demonstrated that NOX4 is upregulated in response to various cardiovascular stressors, including heart failure, myocardial infarction, arrhythmias, and diabetes. This upregulation contributes to detrimental processes like fibrosis, hypertrophy, and inflammation, which are hallmarks of cardiovascular diseases. Inhibition or knockout of NOX4 has shown promise in mitigating these pathological changes, suggesting that NOX4 represents a potential therapeutic target for treating heart disease. However, NOX4’s role is not entirely negative. It also plays a protective role in the heart, supporting myocardial remodeling and angiogenesis and regulating cardiac energy metabolism. Its constitutive ROS production and ability to respond to environmental cues like hypoxia help maintain cellular homeostasis and facilitate adaptive responses to stress. The impact of NOX4 on cardiac health depends not only on its expression level but also on the nature of the stress, the duration of activation, and the balance between protective signaling and oxidative injury. Collectively, the findings suggest that NOX4 functions as a redox sensor, modulating cellular responses to fluctuations in oxidative stress by signaling the need to re-establish redox homeostasis. The ultimate impact of cardiac NOX4 activity, whether protective or deleterious, is highly context-dependent and should not be evaluated through a singular interpretative framework. In conclusion, NOX4 is a dual-function enzyme that can both exacerbate and protect against cardiac pathology, making it a promising, though complex, therapeutic target for various cardiovascular diseases.

## 1. Introduction

“In mythology, the primordial goddess Nox, born from Chaos, represents the night” [1]. This allegorical link between chaos and darkness is echoed in how scientists initially viewed the NADPH oxidase 4 (NOX4) enzyme, which produces reactive oxygen species (ROS) commonly linked to cellular stress and damage. However, just as night also brings rest and renewal within nature balance, NOX4, despite its association with oxidative stress, plays protective and regulatory roles in cardiovascular function, highlighting that darkness is not all destructive.

Experimental and clinical studies indicate that ROS levels rise significantly during heart failure, contributing to its pathophysiology [2]. The increased production of ROS in the stressed heart is thought to result from elevated NOX activity, particularly NOX2 and NOX4, and mitochondrial dysfunction [3,4]. NOX-induced ROS can damage proteins, lipids, and DNA [5], leading to oxidative stress and worsening heart dysfunction. Excess ROS impair cardiomyocyte function and promote maladaptive remodeling like fibrosis, hypertrophy, and apoptosis [6] while also activating pro-inflammatory and stress-related signaling pathways [3,7]. Genome-wide studies have linked the NOX4 locus with homocysteine levels, a known cardiovascular risk factor, in cohorts of Caucasian women [8], European ancestry populations [9] and African Americans [10]. Moreover, genetic variants on NOX4 may affect the impact of homocysteine on cardiovascular mortality [11], further connecting NOX4-mediated oxidative stress to cardiovascular risk.

In their seminal study, Kuroda et al. showed that oxidative stress from NOXs and mitochondria contributes to heart failure, with NOX4-derived ROS playing a key role in fibrosis and inflammation [4]. Animal studies demonstrate that antioxidant therapies [12,13,14] or inhibitory NOX4 targeting [15,16] can mitigate some of the pathological effects of heart failure. These findings suggest therapeutic potential in targeting ROS broadly [17,18,19] or NOX4 specifically [20] for managing heart failure. However, antioxidant trials in heart failure have shown mixed results [21,22], highlighting the need for careful attention to timing, dosage, and target selection. While NOX4 inhibitors have not yet been tested clinically specifically for cardiac disease, preclinical studies suggest benefit in myocardial infarction, heart failure, and ischemia–reperfusion injury. The existing clinical trials for other indications such as liver and pulmonary diseases [23] provide a foundation for considering NOX4 inhibition as a therapeutic strategy in cardiovascular diseases. Conversely, NOX4 may also serve adaptive roles under cellular stress. Several studies have demonstrated that increased NOX4 expression and/or activity can play a protective role in heart diseases by promoting adaptive signaling pathways: these beneficial effects include enhancing cellular survival [24,25], stimulating angiogenesis [26,27,28], modulating autophagy [25] and fatty acid oxidation [29], facilitating stress resilience in cardiac cells and thereby contributing to improved cardiac function and limiting damage under certain pathological conditions.

Several comprehensive reviews have examined the role of NOX4 in the heart, exploring its potentially deleterious [30,31], beneficial [3,32,33] or dual [34] effects on cardiovascular homeostasis, in the context of one or several cardiac pathologies. Collectively, they highlight the need for highly specific therapeutic strategies when targeting NOX4 [35]. However, a key question remains [36]: does increased NOX4 expression contribute directly to detrimental cardiac outcomes, or instead does it reflect a compensatory mechanism aimed at reversing adverse remodeling associated with heart diseases? In other words, is NOX4 induction a driver of oxidative stress or part of an adaptative antioxidant response?

In the present review, we examine preclinical and clinical evidence supporting both the harmful and protective roles of NOX4 in cardiac function, across several major cardiac diseases. The findings suggest that NOX4 may function as a redox sensor, signaling the need to restore homeostatic oxidative balance. Ultimately, whether cardiac NOX4 is beneficial or detrimental largely depends on the context and should not be interpretated through a single lens.

## 2. NOX4 Characteristics

### 2.1. Structure

**Figure 1:**
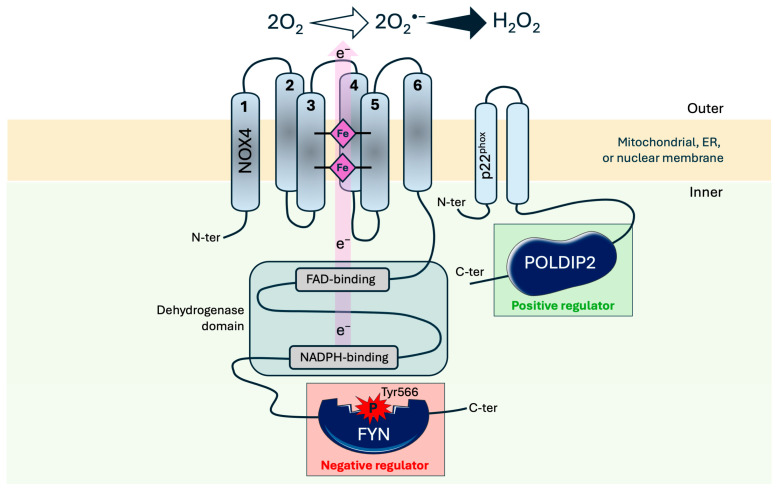
Schematic representation of the NOX4 structure and function. Its activity is positively regulated by the membrane-bound subunit p22^phox^ and by POLDIP2 while negatively regulated by the tyrosine kinase FYN. Numbers 1–6 correspond to the six transmembrane domains of NOX4.

The journey of NOX4 discovery began with a report of a protein homologous to gp91phox (NOX2) in the kidney that was identified as “Renox” [37]. NIH 3T3 fibroblasts overexpressing Renox exhibited increased superoxide (O_2_^•^) production and signs of cellular senescence, highlighting Renox/NOX4 involvement in redox signaling [37]. Then, the cloning of human NOX4 cDNA confirmed that it was a novel member of the NOX family, consisting of 578 amino acids and sharing significant homology with other NOXs, specifically 39% identity to NOX2 and 35% to NOX1 [38]. NOX4 is experimentally confirmed to span the membrane six times, with both its N- and C-termini facing the cytosol; the first, third, and fifth extracellular loops are predicted to be luminal, whereas the second and fourth loops face the cytosolic side [39] (Figure 1). The C-terminal cytosolic region contains the dehydrogenase domain, which includes FAD-binding and NADPH-binding lobes responsible for intracellular electron transfer. The electrons are then relayed to heme groups coordinated within specific transmembrane helices (notably the third and fifth transmembrane helices), ultimately reducing molecular oxygen [40] (Figure 1).

NOX4 is a unique isoform of the NOX enzyme family, playing a pivotal role in signaling within cardiomyocytes. Indeed, unlike other NOX enzymes that primarily generate O_2_^•^, NOX4 predominantly produces hydrogen peroxide (H_2_O_2_), a relatively stable and membrane-permeable ROS [41,42]. In addition, NOX4 differs from other NOX isoforms that require the assembly of several cytosolic regulatory subunits such as p47^phox^, p67^phox^, or Rac, to generate ROS [43]. Indeed, NOX4 is constitutively active, is associated with the membrane-bound subunit p22^phox^, but it does not need additional cytosolic subunits for producing ROS [42,43,44] (Figure 1). The importance of p22^phox^ in NOX4 function was further confirmed by Prior et al. who used CRISPR/Cas9 technology to knockout p22^phox^ in HEK293 cells expressing NOX4: they showed that p22^phox^ was essential for the maturation of NOX4 and its ability to produce H_2_O_2_, as its knockout ablated NOX4 activity [45].

### 2.2. Alternative Splicing

The study of NOX4 has revealed the existence of multiple splice variants, each with unique structural and functional characteristics that contribute to the diverse roles of the enzyme in cellular signaling and stress responses. One of the earliest studies to investigate these isoforms was conducted by Goyal et al. [46], who identified several splice variants of NOX4, including NOX4B, NOX4C, NOX4D, and NOX4E, in the human lung A549 cell line and lung tissues. Notably, they found that NOX4B lacks the first NAD(P)H binding site, while NOX4C lacks both the NAD(P)H and FADH binding sites [46]. These alterations resulted in a decrease in ROS levels in cells overexpressing NOX4B or NOX4C, suggesting that these isoforms exhibit dominant-negative characteristics, effectively inhibiting ROS production. In contrast, NOX4D and NOX4E, which lack transmembrane domains, are non-membrane associated isoforms [46]. Interestingly, NOX4D, despite lacking a transmembrane domain, contained all the necessary binding sites for FADH and NAD(P)H, and exhibited ROS production rates similar to the full-length NOX4 protein, highlighting its functional resemblance to the canonical form of NOX4 [46].

Building on this, Anilkumar et al. [47] focused on the NOX4D splice variant, a 28-kDa isoform. Their research showed that in vascular cells, NOX4D significantly enhances NADPH-dependent ROS production, and increases the phosphorylation of key signaling molecules, such as Erk1/2 and Elk-1, suggesting that NOX4D plays a critical role in cellular signaling pathways. Furthermore, the overexpression of NOX4D was shown to induce DNA damage, as evidenced by increased γ-H_2_AX phosphorylation, a marker of DNA double-strand breaks. These findings suggest that NOX4D, through its elevated ROS production, may be involved in cellular stress responses and the signaling pathways that regulate DNA damage and repair [47].

The importance of NOX4 splice variants extends beyond lung and vascular cells, as a more recent work has also highlighted their relevance in cardiac disease. Varga et al. [48] examined the role of alternative splicing of the NOX4 gene in heart failure using deep RNA sequencing of cardiac transcriptomes. Their study revealed extensive alternative splicing of NOX4 in heart failure samples, compared to healthy donor hearts. Importantly, they found that the full-length NOX4 isoform was significantly upregulated in ischemic cardiomyopathy, suggesting that NOX4 contributes to ROS production in the context of heart failure. These findings support the notion that alternative splicing of NOX4 may regulate its function in the pathogenesis of cardiac diseases, particularly in the generation of ROS during heart failure [48].

Together, these studies underscore the complexity of NOX4 regulation through alternative splicing, revealing isoforms with distinct roles in ROS production, cellular signaling, and stress responses. The functional diversity of NOX4 splice variants, from their influence on ROS generation to their potential involvement in DNA damage signaling and heart failure, emphasizes the importance of understanding the specific roles of each isoform in different cellular contexts and disease states.

### 2.3. Functional Regulation

The functional regulation of NOX4 in different cellular contexts has become a significant area of interest. For instance, FYN, a Src family kinase, has been reported to colocalize with NOX4 in perinuclear mitochondria, endoplasmic reticulum (ER), and nuclear fractions in neonatal rat ventricular cardiomyocytes [49]. Through biochemical assays, this latter study demonstrated that FYN interacts with the C-terminal domain of NOX4 and phosphorylates tyrosine 566 (Y566), leading to decreased NOX4 activity (Figure 1). This phosphorylation acts as a negative feedback regulation, reducing ROS production, which is crucial in preventing pathological hypertrophy in the heart [50]. FYN was identified as the first direct negative regulator of NOX4 through post-translational modification, providing novel insight into how NOX4 activity is modulated in heart cells [49]. In contrast, Poldip2 was shown to act as a positive regulator of NOX4, by binding to its subunit p22^phox^ to activate NOX4 (Figure 1) in vascular smooth muscle cells (VSMCs) [51]. These findings underscore the dual nature of NOX4 regulation, where proteins, such as FYN and Poldip2, can either inhibit or enhance NOX4 activity, depending on the cellular context to maintain a fine balance in NOX4-derived ROS for cellular homeostasis and tissue health. The regulation of NOX4 activity is also tightly linked to its expression levels. In a tetracycline-inducible NOX4 expressing cell line, a strong correlation between NOX4 mRNA levels and ROS production was found [52]. This suggests that NOX4 activity is primarily regulated by its transcription, making its mRNA expression a key determinant of its ROS-generating capacity [52]. However, more recently, it has been proposed that since NOX4 is constitutively active, H_2_O_2_ production is controlled by its protein abundance and not at the transcriptional level [53]. Indeed, in rat H9c2 cardiomyocytes, hypoxia upregulated NOX4 protein levels and H_2_O_2_ production, despite no change in NOX4 mRNA expression; this increase in NOX4 protein was dependent on p22^phox^ [53].

### 2.4. Subcellular Localization in the Heart

The expression of NOX4 in cardiac tissues was first explored by Colston et al., who demonstrated that adult cardiac fibroblasts express both the mRNA and protein for NOX4, along with its associated subunit p22^phox^ [54]. The study of NOX4 expression across different vascular tissues further emphasizes its significance: NOX4 is expressed at much higher levels than NOX2 in VSMCs [55]. NOX4 expression was also found in human endocardial endothelial cells, human vascular endothelial cells and VSMCs [56], underlining the potential role of NOX4 in vascular function. Furthermore, the presence of NOX4 in human cardiomyocytes, VSMCs, endothelial cells, and fibroblasts was confirmed [57]. This highlights the broad distribution of NOX4 in cardiac and vascular tissues. In line with this, Zou et al. [58] used sequential comprehensive bioinformatics analysis of human sepsis cardiac transcriptome data to confirm that NOX4 is expressed in cardiomyocytes as well as other cell types, including immune cells, both in fetal and adult human hearts. This study further supports the idea that NOX4 plays a central role in heart function and its pathological alterations, particularly in the context of septic myocardial injury.

The subcellular localization of NOX4 contributes significantly to its functional versatility. In human endothelial cells, NOX4 is expressed at levels significantly higher than NOX2, with NOX4 predominantly localized to the ER [59]. This unique subcellular localization differentiates NOX4 from other NOXs, which are typically found in the plasma membrane or mitochondria. The finding that NOX4 does not overlap with lysosomes, mitochondria, or Weibel-Palade bodies suggests its distinct role within the ER in endothelial cells [59]. On the ongoing debate over NOX4 localization, the majority of the studies confirmed its localization in the ER of cardiomyocytes [25,28,60], whereas some failed to identify NOX4 in pure cardiac mitochondria in basal conditions [61]. NOX4 levels were also reported to be significantly increased in the ER of VSMCs from spontaneously hypertensive rats (SHR), and no NOX4 was detected in isolated mitochondria [62]. However, treatment with mitoTEMPO, a mitochondrial antioxidant, reduced basal ROS levels in SHR cells, suggesting that while primary localization and action of NOX4 occur within the ER, NOX4 activity may also be linked to mitochondrial ROS under certain conditions [62].

In contrast, NOX4 was found predominantly localized in mitochondria in both cultured cardiac myocytes and in transgenic NOX4 mouse hearts, highlighting that NOX4 could contribute to mitochondrial ROS production [63]. During conditions of heightened NOX4 expression, such as hypoxia, NOX4 may translocate to mitochondria, contributing to mitochondrial ROS production [61].

The subcellular localization of NOX4 has been refined by providing compelling evidence that NOX4 is enriched in the ER-mitochondria contact sites, known as mitochondria-associated membranes, in cardiac cells [24]. The use of subcellular fractionation and electron microscopy confirmed NOX4 involvement in inter-organellar signaling and ROS production within the heart, particularly at the junctions between the ER and mitochondria, where it may play a role in regulating cellular oxidative stress [24].

Altogether, these data suggest that cardiac NOX4 expression are highly context-dependent, varying across the type of cardiac stress, cell types and subcellular compartments.

## 3. Physiological Roles and Stress-Responsive Regulation of NOX4 in the Heart

### 3.1. NOX4 in Cardiac Differentiation and Development

NOX4-derived ROS play a pivotal role in cardiomyocyte differentiation and cardiac development: NOX4 is the principal NOX isoform expressed in mouse undifferentiated embryonic stem cells (ESCs), embryoid bodies (EBs), and neonatal cardiomyocytes [64]. Its downregulation in murine ESCs and embryonal carcinoma cells has been shown to impair cardiogenesis, highlighting its essential role in early cardiac development [64,65,66,67,68]. Mechanistically, NOX4 supports cardiac lineage commitment via redox-sensitive activation of signaling pathways fundamental to the cardiac differentiation process such as c-Jun/GATA-4 [67] and p38 MAPK/MEF2C (myocyte enhancer factor 2C) [64]. Moreover, in a transgenic mouse model overexpressing NOX4, enhanced cardiomyocyte proliferation was observed, dependent on ERK1/2–c-Myc signaling, suggesting a possible role for NOX4 in cardiac regeneration [69]. However, some findings complicate the view of NOX4 as a critical player in cardiomyocytes differentiation and development. Indeed, Crescini et al. (2013) reported that NOX4 expression increased during ESCs differentiation regardless of whether cardiomyocyte formation occurred [70], suggesting that NOX4 upregulation alone may not predict successful cardiomyocyte differentiation. These discrepancies underline the need for further studies to clarify NOX4 developmental role in varying cellular contexts.

### 3.2. NOX4 in Angiogenesis and Endothelial Adaptation

NOX4 also plays a critical role in promoting angiogenesis, particularly in endothelial progenitor cells. In cord blood-derived endothelial colony-forming cells (CB-ECFCs), NOX4 activation by phorbol esters enhanced angiogenic activity, while its knockdown significantly impaired tube formation [26]. Under hypoxic conditions, reduced NOX4 signaling in CB-ECFCs was associated with diminished angiogenic capacity, which was partially restored by NOX4 overexpression [27]. This rescue was linked to H_2_O_2_ production and nuclear factor erythroid 2–related factor 2 (Nrf2) activation, a transcription factor that plays a key role in regulating the expression of antioxidant proteins and genes involved in cellular defense against oxidative stress [27]. These findings suggest that NOX4 supports vascular adaptation in ischemic environments.

### 3.3. NOX4 in Cardiac Homeostasis and Resistance to Acute Stress

Beyond development, NOX4 contributes to cardiac homeostasis (Table 1). Transgenic mice with cardiomyocyte-specific NOX4 overexpression display increased expression of Nrf2-regulated antioxidant genes, enhanced cell cycle activity, and greater proliferative capacity in cardiomyocytes [69,71] (Table 1). Accordingly, NOX4 knockdown in neonatal cardiomyocytes or cardiomyocyte-specific NOX4 deletion in mice subjected to acute exercise resulted in impaired cardiac function and reduced antioxidant gene expression [72] (Table 2).

Importantly, NOX4 modulates cardiac metabolism under stress. In transgenic mice, NOX4 overexpression induced a metabolic shift characterized by enhanced fatty acid oxidation and reduced glucose oxidation, partly via O-GlcNAcylation-mediated pathways [29] (Table 1). This adaptation was associated with improved contractile function under pressure overload and reversed by NOX4 downregulation (Table 2). Notably, endothelial-specific NOX4 overexpression led to physiological cardiac hypertrophy with preserved function, suggesting broader roles for NOX4 in cardiovascular remodeling [73].

### 3.4. NOX4 as a Redox Sensor in Response to Environmental Stress

Recent studies have uncovered how external factors like cellular stress can influence NOX4 activity, positioning NOX4 as a redox-sensitive sensor that orchestrates adaptive responses to environmental stress. In rat H9c2 cardiomyocytes under hypoxic conditions, the protein dimer NOX4-p22^phox^ was stabilized: hypoxia decreased the degradation (removal) of NOX4 by autophagy, leading to stabilization and accumulation of NOX4 protein and H_2_O_2_ production [53]. Hypoxia-induced NOX4 expression is hypoxia-inducible factor 1-alpha (Hif1α)-dependent, promoting cellular proliferation in pulmonary smooth muscle cells [82]. Conversely, in mice with systemic or cardiac-specific NOX4 deletion, lower ROS levels reduced Hif1α, worsening ischemia/reperfusion (I/R) injury [81]. This suggests that NOX4 may function as a physiological sensor involved in myocardial adaptation [81]. In response to pressure overload, NOX4 upregulation promotes angiogenesis via activation of Hif1α and vascular endothelial growth factor (VEGF) release, preserving myocardial capillary density [28]. In contrast, lower cardiac NOX4 protein levels observed in streptozotocin-treated diabetic rats correlate with impaired angiogenesis [83]. Under energy stress conditions such as glucose deprivation, NOX4-driven ROS induced autophagy to protect cardiomyocytes and increase their survival by favoring the turnover of damaged proteins and dysfunctional organelles [25]. Conversely, NOX4 knockdown reduced cardiomyocytes viability [25]. Therefore, whereas hypoxia stabilizes NOX4 by decreasing its autophagic degradation in cardiomyocytes, NOX4-driven ROS induces autophagy to protect cardiomyocytes during energy stress. This suggests a dynamic interplay where NOX4 protein levels and autophagy mutually influence each other depending on the type and duration of cellular stress. This also suggests that NOX4 plays a protective role during prolonged ischemia and fasting, by supporting adaptive survival mechanisms.

**Figure 2:**
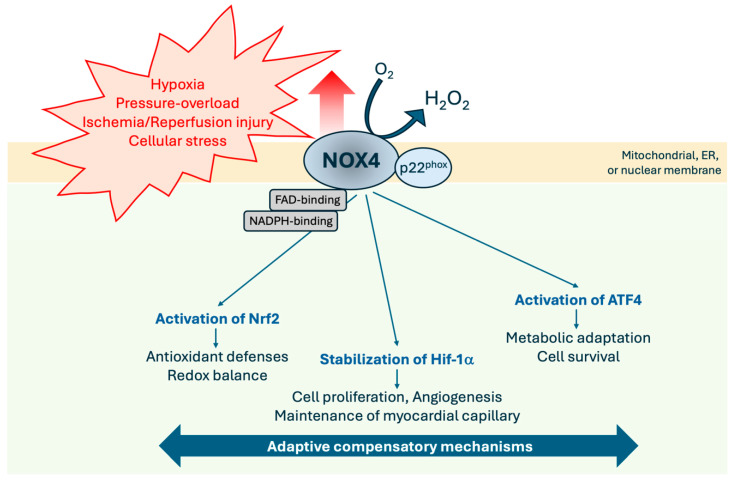
Schematic representation of adaptive compensatory mechanisms activated by upregulation of NOX4 under cellular stress.

In the presence of cellular stress, NOX4 activates key transcription factors involved in stress adaptation. These include the activating transcription factor 4 (ATF4), which regulates antioxidant, amino acid, and autophagy pathways [25,29,32], and Nrf2, a master regulator of antioxidant gene expression, activated by NOX4-derived H_2_O_2_ [71,84] (Figure 2). Thus, NOX4 supports cellular adaptation through the regulation of redox-sensitive transcription factors, and together, these pathways mediate metabolic reprogramming, antioxidant defense and cell survival during acute and chronic stress. Given its broad roles in both cardiac and vascular physiology, NOX4 represents a promising therapeutic target for enhancing myocardial resilience and promoting repair in cardiovascular disease.

Collectively, the evidence positions NOX4 as a central regulator of cardiac development, homeostasis, and adaptation to stress. Through its tightly regulated ROS production and redox-sensitive signaling, NOX4 influences key adaptive pathways leading to cardiomyocyte differentiation, proliferation, angiogenesis, antioxidant defense, and metabolic remodeling. In response to dynamic environmental stressors, NOX4 may therefore function as a physiological sensor, orchestrating cardiac protective responses to hypoxia, ischemia, energy deprivation, and pressure overload (Figure 2). NOX4 dual capacity to promote either protective or pathological effects is context-dependent, i.e., shaped by the type, intensity and duration of stress, as well as its subcellular localization and interaction with transcriptional networks (see below, Section 5).

## 4. NOX4 in Heart Diseases

Despite extensive research, the specific role of NOX4 in cardiac diseases remains controversial. On the one hand, increased endogenous NOX4 expression and/or activity has been observed in a large number of animal models of heart dysfunction (induced by surgery or chemicals), as well as in cardiac cell models, including primary cardiomyocytes (neonatal or adult, from mouse or rat), iPSCs-derived cardiomyocytes and cardiac cell lines (H9c2, HL-1 and AC16) subjected to stress or chemical treatment. These findings suggest a pathological role for NOX4 (Table 3). On the other hand, and more recently, NOX4 has emerged as an adaptative protein involved in the cellular stress response, with increased NOX4 expression and/or activity linked to beneficial effects in the context of cardiac diseases (Table 1). In the present review, we focus on preclinical studies involving models of cardiac diseases in which NOX4 expression was specifically induced and/or repressed using transgenic approaches, adenoviruses or chemicals, to demonstrate causality between upregulation of NOX4 and cardiac effects, either deleterious or beneficial. The present chapter focuses exclusively on the mechanistic role of NOX4 in cardiac pathophysiology, drawing on evidence from transgenic and knockout models that establish its causal involvement in deleterious versus adaptive responses. In contrast, therapeutic approaches aiming to inhibit or silence NOX4, including siRNA/shRNA strategies and pharmacological interventions, are presented separately in Section 6, where the potential of NOX4 as a therapeutic target is discussed.

### 4.1. Deteleterious Role of Induced NOX4 in Cardiac Pathologies

#### 4.1.1. Contribution of Induced NOX4 in Heart Failure

In mice exposed for 2 weeks of transverse aortic constriction (TAC; aortic arch) [4,49], or continuous infusion of phenylephrine [60], specific transgenic cardiac overexpression of NOX4 worsened cardiac outcomes. Specifically, NOX4 overexpression exacerbated left ventricular (LV) contractile dysfunction and cardiac hypertrophy following TAC [4,49], and induced adverse cardiac remodeling to phenylephrine [60] (Table 3). These findings indicate that NOX4 upregulation aggravates maladaptive cardiac responses under chronic pressure overload or continuous adrenergic stress. Importantly, the detrimental role of NOX4 has also been demonstrated at the mitochondrial level: transgenic mitochondrial-specific NOX4 overexpression in mice provoked mitochondrial damage and diastolic dysfunction associated with increased myocardial fibrosis, and altered calcium influx and Z-disc structure [85] (Table 3). These findings highlight mitochondria as a critical compartment where NOX4 contributes to structural and functional deterioration of the heart.

Conversely, loss-of-function approaches consistently demonstrate protective effects. Cardiac-specific deletion of NOX4 in transgenic mouse models subjected to TAC led to improved LV contractile function and a reduction in cardiac hypertrophy, fibrosis and apoptosis [4,60] (Table 4). Similarly, when these NOX4-deficient mice were exposed to continuous phenylephrine infusion, they exhibited less cardiac hypertrophy and oxidative stress [60]. Additional models support this protective effect of NOX4 deletion: systemic deletion of NOX4 in mice subjected to aortocaval shunt limited LV dilatation and hypertrophy [96]. Transgenic cardiac-specific deletion of NOX4 in FYN-knockout mice subjected to TAC rescued the exaggerated cardiac hypertrophy, reduced cardiac ROS production and apoptosis [49]. In Elmo1-hypermorphic mice, systemic NOX4 deletion alleviates the dilated cardiomyopathy phenotype, restores cardiac morphology and LV contractile function [97] (Table 4). In response to isoproterenol, NOX4 deletion also resulted in preserved diastolic function along with reduced cardiac inflammation and fibrosis [98] (Table 4).

In vitro, overexpression of NOX4 further highlights its detrimental role. In cardiac cells stimulated with doxorubicin or isoproterenol, NOX4 upregulation aggravated oxidative stress and apoptosis [50,89,90] (Table 3). Neonatal rat cardiomyocytes overexpressing NOX4 also exhibited increased apoptosis [63] and expression of hypertrophic markers [88], whereas H9c2 cells overexpressing NOX4 demonstrated increased cell death [86] and cytokine production [87] (Table 3).

It was reported that in patients undergoing coronary artery bypass graft (CABG) surgery, NOX4 expression in the right atria was significantly higher in those with reduced left ventricular ejection fraction (LVEF ≤ 45%) compared to patients with preserved LVEF (>45%) [125]. Accordingly, NOX4 expression was 3-fold increased in the LV from advanced heart failure patients compared to non-failing controls [99]. Increased expression of NOX4 was also associated with mitochondrial oxidative stress and dysfunction in LV myocardial samples with increased fibrosis and myofibroblast activation, from patients with a documented history of moderate diastolic dysfunction [85]. In failing hearts obtained from patients with dilated cardiomyopathy (compared to donors with normal hearts), the level of NOX4 was significantly higher, whereas the level of NOX4 phosphorylated at Y566 was lower, resulting in a decreased ratio of phosphorylated NOX4 to total NOX4 in dilated cardiomyopathy (DCM) hearts [49]. NOX4 expression was increased in human myocardial specimens collected from explanted hearts of non-ischemic DCM patients during cardiac transplantation (compared to brain-dead donor hearts with no history of heart disease), and its expression was positively associated with the cleavage of caspase-1 and gasdermin D expression, two effectors of pyroptosis, a type of programmed cell death that is closely associated with inflammation [126]. Bioinformatic analysis of the transcriptional profiles of human failing hearts, obtained from patients with end stage DCM who underwent transplantation, identified NOX4 as one of six co-regulated genes (with *POSTN*, *CTGF*, *FN1*, *LOX* and *TGFB2*) with established link to heart failure development [127]. Gene expression profile from the Gene Expression Omnibus (GEO) database showed that NOX4 expression was elevated in human DCM myocardial samples, connecting with immune response and ferroptosis, a unique type of programmed cell death that depends on iron and is driven by lipid peroxides [128]. NOX4 expression was also increased in human cardiac fibroblasts isolated from failing left ventricles compared to normal controls [116].

In contrast, Borchi et al. reported that NOX4 mRNA levels were unchanged in explanted failing hearts obtained from patients undergoing transplantation for end-stage heart failure secondary to idiopathic dilated cardiomyopathy, despite enhanced superoxide production by NOXs in these failing ventricles [129]. Finally, Moreno et al. [57] suggested a potential role of NOX4 deficiency in adverse heart remodeling, as myocardial NOX4 levels were abnormally decreased in patients with severe aortic valve stenosis (consequent to pressure-overload), and correlated positively with parameters of LV systolic function, i.e., ejection fraction and fractional shortening, in these patients.

In summary, experimental studies in mice indicate that NOX4 induction aggravates cardiac dysfunction and pathological remodeling in heart failure, while loss-of-function approaches consistently mitigate these deleterious effects. In human, most studies have reported increased NOX4 expression in failing hearts, but whether this rise reflects a causal pathogenic role or a compensatory antioxidant response remains unresolved.

#### 4.1.2. Contribution of Induced NOX4 in Myocardial Infarction

In Sprague-Dawley rats with left anterior descending (LAD) coronary artery ligation, NOX4 adenoviral overexpression abrogated the beneficial effects of endostatin and Tanshinone IIA on myocardial ischemia (MI), and exacerbated LV systolic dysfunction and myocardial fibrosis [92,93] (Table 3). Similarly, in mice exposed to LAD ligation, NOX4 mRNA and protein levels gradually increased postoperatively (from 6 h to 14-day) in LV tissues; the expression of NOX4 positively correlated with oxidative stress level in MI mice, suggesting that NOX4 contributes to the pathology [107]. Conversely, systemic NOX4 deletion in transgenic mice subjected to LAD coronary artery ligation decreased infarct area and myocardial adverse remodeling [87] (Table 4). Finally, hearts from patients with ischemic cardiomyopathy showed increased NOX4 expression and oxidative stress, compared to control nonfailing hearts [87]. Interestingly, in these patients, NOX4 protein expression was positively correlated with myocardial cell death, LV fibrosis, cardiomyocyte size and inflammatory cytokines expression [87]. Together, these findings indicate that NOX4 aggravates myocardial injury after infarction.

#### 4.1.3. Contribution of Induced NOX4 in Ischemia/Reperfusion Injury

Some studies reported a deleterious effect of NOX4 on ischemia/reperfusion (I/R) outcome: transgenic mice with cardiac specific overexpression of NOX4 exhibited increased infarct size, and decreased cardiac energetics and contractile performances after exposure to I/R [80] (Table 3). Consistently, loss-of-function approaches support a protective effect of NOX4 suppression: systemic and cardiac-specific deletion of NOX4 in mice subjected to I/R decreased infarct size/area at risk, myocardial apoptosis and O_2_^•^ production [81] (Table 4). In vitro, knockdown of NOX4 in neonatal cardiomyocytes subjected to hypoxia/reoxygenation increased cell viability, further confirming its deleterious contribution [81] (Table 4). Altogether, evidence from both in vivo and in vitro models indicates that NOX4 upregulation aggravates I/R-induced cardiac injury, whereas its deletion in transgenic mice preserves myocardial survival and function.

#### 4.1.4. Contribution of Induced NOX4 in Atrial Fibrillation (AF) and Arrythmia

In zebrafish embryo, injection of human NOX4 RNA at one-cell stage provoked a phenotype of irregular heartbeats, with increased (superoxide-dependent) H_2_O_2_ production and calcium/calmodulin-dependent protein kinase II (CaMKII) activation, providing direct in vivo evidence for a causal role of NOX4 in arrhythmic phenotypes [94] (Table 3).

In humans, NOX4 upregulation has also been consistently linked to atrial dysfunction. Zhang et al. [130] reported that H_2_O_2_ production was more than doubled in AF patients, and this increase correlated with a twofold upregulation in NOX4 expression. Likewise, Kang et al. [106] investigated the differences in gene expression between patients with AF and patients with sinus rhythm by analyzing three independent datasets from GEO, and demonstrated that NOX4 expression was upregulated in AF and associated with cardiac hypertrophy-related genes.

Altogether, evidence from zebrafish and human studies suggest that NOX4 upregulation promotes oxidative stress, calcium handling abnormalities, and structural remodeling, thereby facilitating the development of cardiac arrhythmias, particularly AF. This consistent association highlights NOX4 as a potential therapeutic target in AF and related arrhythmogenic conditions.

#### 4.1.5. Contribution of Induced NOX4 in Other Heart Diseases

In heart valve diseases

Hagler et al. demonstrated that NOX4 expression was increased in mitral valves from humans with myxomatous mitral valve disease, in whom LV function was well maintained [131]. In contrast, a study suggested that NOX4 deficiency may contribute to adverse myocardial remodeling, as NOX4 expression was abnormally reduced in patients with severe aortic valve stenosis and pressure overload [57]. Furthermore, NOX4 levels were positively associated with LV systolic function parameters, including ejection fraction and fractional shortening [57], suggesting a beneficial role of NOX4.

In congenital heart diseases

It has been reported that protein levels of NOX4 were elevated in myocardial tissue samples obtained from fetuses with congenital heart disease [132]. This increase was associated with upregulation of the ferroptosis marker ACSL4, downregulation of antioxidant proteins NRF2, GPX4, and HO-1, and elevated levels of malondialdehyde, a marker of lipid peroxidation [132].

In age-related heart failure

Interestingly, Ago et al. showed that cardiac NOX4 expression increased gradually between 3 and 12 months in mice [63] (Table 3). Upregulation of NOX4 was diffusely observed in the myocardium, including in cardiac myocytes, in response to aging. Moreover, aged (13–14 months) transgenic mice with cardiac-specific overexpression of NOX4 exhibited LV contractile dysfunction with increased myocardial fibrosis, apoptosis, oxidative stress and mitochondrial dysfunction, with no obvious cardiac hypertrophy [63].

In myocarditis

NOX4 was identified as a positive regulator of ferroptosis in 20 hearts sourced from patients who died from sepsis (GSE79962 from the GEO database) compared to 11 control hearts from non-failed donors [58]. This insight was validated in an in vivo septic myocardial injury model, i.e., BALB/c mice injected intraperitoneally with lipopolysaccharide [58].

In Duchene muscular dystrophy

In a mouse model of human Duchene muscular dystrophy (Mdx mice), deletion of Cardiac Isl1-interacting Protein (CIP) induced dystrophic cardiomyopathy and heart failure [95] (Table 3). Overexpression of NOX4 further accelerated cardiomyopathy progression in these Mdx mice. In contrast, increased NOX4 expression alone had no significant effect on cardiac dimensions, fibrosis, or marker gene expression in control mice [95]. Together, these findings suggest that NOX4 contributes to the development of cardiomyopathy in the absence of dystrophin, underscoring its pathogenic role in dystrophic heart disease.

Overall, these preclinical and clinical findings demonstrate the pathological roles of NOX4 in various heart diseases, where its increased activity contribute to disease progression, thereby supporting NOX4 as a promising therapeutic target.

### 4.2. Beneficial Role of Induced NOX4 in Cardiac Pathologies

As introduced earlier, cardiac NOX4 plays beneficial roles, particularly under physiological or compensatory conditions (Figure 2). In addition, in experimental models of cardiac diseases, overexpression of cardiac NOX4 has also been shown to improve cardiac function, both in vivo and in vitro (Table 1).

#### 4.2.1. Beneficial Contribution of Induced NOX4 in Heart Failure

Some studies reported that NOX4 could be a physiological effector in response to pathological stimuli, thus alleviating cardiac dysfunction and adverse remodeling (Table 1 and Table 2). In transgenic mice subjected to TAC surgery (suprarenal banding) for 9 weeks, cardiac-specific overexpression of NOX4 was reported to preserve cardiac function and decrease cardiac hypertrophy and fibrosis, by increasing angiogenic markers expression and fatty acid oxidation [28,29] (Table 1). Moreover, endothelial-specific NOX4 overexpression in mice protects against myocardial fibrosis, inflammatory cell infiltration and endothelial activation induced by angiotensin II insult [73]. Accordingly, both systemic [28,75] or specific deletion of NOX4 in cardiomyocytes or endothelium [75] demonstrated exaggerated cardiac dysfunction, hypertrophy and adverse remodeling following hemodynamic overload induced by TAC surgery (aortic arch or suprarenal banding) (Table 2). The disruption of NOX4 by CRISPR-Cas9 genome editing in H9c2 cells exacerbated the decrease in cell viability provoked by acrolein or methyl vinyl ketone insult [78] (Table 2). Finally, cardiomyocytes isolated from transgenic systemic NOX4-knockout mice prevented insulin-induced attenuation of cardiomyocyte contractility and β-adrenergic signaling activity after isoproterenol stimulation [79] (Table 2). Altogether, this data suggests that NOX4 is induced, in the adult heart, as an adaptive stress response to pathophysiological insult.

#### 4.2.2. Beneficial Contribution of Induced NOX4 in MI

Cardiac-specific overexpression of NOX4 increased survival rate by preserving LV systolic and diastolic function, and decreased cardiac hypertrophy, fibrosis and metalloproteinase activity, in transgenic mice subjected to left coronary artery ligation [74] (Table 1). Mechanistical investigations revealed that in both neonatal rat cardiomyocytes and H9c2 cells subjected to hypoxia, adenoviral NOX4 overexpression increased angiogenic markers expression, suggesting that NOX4 could act as a key determinant of cardiac adaptation to ischemic stress [28] (Table 1). Accordingly, Sciarretta et al. [25] demonstrated that during fasting and prolonged ischemia, NOX4 was activated in the mouse heart, and that cardiac-specific NOX4-knockout mice exposed to energy stress exhibited increased infarct size and myocardial necrosis, and decreased LV contractile function, myocardial ATP content and autophagy activation, suggesting that cardiac NOX4 critically mediates autophagy in response to energy stress to ensure cardioprotection (Table 2).

#### 4.2.3. Beneficial Contribution of Induced NOX4 in I/R

One team demonstrated that NOX4 improved I/R outcome: in transgenic mice subjected to I/R, cardiac-specific overexpression of NOX4 preserved LV systolic function and increased macrophages proportion and polarization toward an M2 phenotype, resulting in improved post-MI survival, remodeling and healing [74] (Table 1). Conversely, in systemic NOX4-KO mice, in Langendorff-perfused hearts subjected to I/R, infarct size and ER stress-associated cell death increased, together with a lower recovery of contractile function after reperfusion [24,77] (Table 2). These data point to a crucial role for NOX4 in enhancing cell survival after ischemic stress.

Interestingly, some findings mitigate these insights: Yu et al. [80] demonstrated that in a Langendorff system, heart from transgenic mice with cardiac-specific overexpression of NOX4 and subjected to I/R, exhibited increased infarct size, impaired cardiac energetics and decreased contractile performance (Table 2). Surprisingly, they also reported that cardiac-specific expression of a dominant-negative NOX4 (with a Phe437His mutation, which competes with both NOX4 and NOX2) also exacerbated I/R injury [80,81] (Table 2). Accordingly, although isoform-specific genetic deletion of either NOX2 or NOX4 significantly attenuates I/R injury in the heart, combined deletion of systemic NOX2 and cardiac-specific NOX4 paradoxically aggravated infarct size and myocardial apoptosis in mice [81] (Table 2). Moreover, cardiac-specific NOX4 overexpression in mice subjected to I/R did not alter infarct size or apoptosis despite increased O_2_• production [81]. Finally, it was also reported that systemic deletion of NOX4 in mice subjected to I/R had no effect on the infarct size/area at risk [133], contrasting with the findings discussed above [81]. Together, these contradictory observations underscore the complexity of NOX-derived ROS in I/R injury, and the underlying mechanisms are further discussed in Section 5.

#### 4.2.4. Beneficial Contribution of Induced NOX4 Atrial Fibrillation and Arrhythmia

To the best of our knowledge, there are no study showing that NOX4 is beneficial in AF or arrythmia.

In summary, the role of NOX4 in cardiac pathophysiology is complex. Although NOX4 overexpression in response to pathological stimuli (such as pressure overload, hypoxia, or I/R injury) can exacerbate oxidative stress, fibrosis and cardiac dysfunction, several studies using transgenic mice have shown that NOX4 can also play protective roles under certain conditions. Both excessive and insufficient NOX4 activity can be detrimental, particularly in I/R injury, where maintaining a balanced redox environment appears critical. This dual nature suggests the existence of a “threshold” level of NOX4-derived ROS that determines whether its effects are adaptive or harmful [34]. Further research is essential to delineate the precise conditions under which NOX4 exerts protective versus pathological effects, to inform context-specific therapeutic strategies.

## 5. Evidence Explaining the Discrepancies of Deleterious and Beneficial Cardiac Effects of NOX4

The literature more often reports NOX4 activity as detrimental (Table 3) than protective (Table 1). Most of this evidence comes from loss-of-function approaches such as knockout murine models, siRNA or shRNA-mediated knockdown (Table 2 and Table 4) or pharmacological inhibitors with varying specificity (see below, Section 6), rather than from transgenic overexpression (Table 1 and Table 3). To robustly establish the causal role of NOX4 in cardiac health, both direct approaches (such as transgenic overexpression) and complementary loss-of-function strategies (such as gene silencing or pharmacological inhibition), should ideally be conducted within the same study by the same research team. For obvious reasons, this comprehensive approach is rarely undertaken, even in simpler experimental systems like cultured cells. This methodological limitation partly explains why distinct groups, using seemingly similar strategies in comparable models, often reach opposing conclusions.

This issue is well illustrated in heart failure models. Cardiac-specific overexpression of NOX4 in transgenic mice led to aggravated cardiac dysfunction and hypertrophy after 2 weeks of pressure overload [4,49] (Table 3), while cardiac-specific NOX4 knockout under the same stimulus showed protective effects (Table 4), overall suggesting a detrimental role for NOX4. In contrast, other studies reported that cardiac-specific NOX4 overexpression preserved cardiac function and reduced hypertrophy after 9 weeks of TAC, whereas systemic or cardiac-specific NOX4 deletion in that context worsened cardiac remodeling [28,29] (Table 1 and Table 2), pointing instead to a protective role for NOX4. These discrepancies likely stem from multiple interrelated factors. A minor contribution may arise from differences in mouse strain (FVB vs. C57BL/6), but more decisive variables include the duration (2 weeks vs. 9 weeks) and severity (proximal aortic constriction vs. milder transverse aortic constriction) of the TAC protocols, as well as differences in transgene design. For instance, targeting the translation initiation site and the first two exons may abolish NOX4 expression entirely [28], whereas disruption of a downstream exon may result in the expression of truncated NOX4 forms with residual activity [4].

A similar pattern of context dependence emerges in I/R models. Short reperfusion (25 min ischemia/1 h reperfusion) in NOX4-overexpressing mice led to increased infarct size and impaired energetics [80] (Table 3), whereas longer reperfusion (30 min ischemia/3–7 days reperfusion) in a similar model improved cardiac function and promoted reparative M2 macrophage polarization [74] (Table 1). In this case too, the length and context of the ischemic stimulus appear to determine whether NOX4 exerts deleterious or adaptive effects.

Even more strikingly, inconsistencies can arise within the same study under identical conditions. Matsushima et al. [81] (Table 2 and Table 4) demonstrated that isoform-specific deletion of either NOX2 or NOX4 significantly reduced infarct size, apoptosis, and superoxide production in mice subjected to 30 min ischemia followed by 24 h reperfusion. In contrast, combined deletion of both isoforms—either through systemic NOX2 knockout with cardiac-specific NOX4 knockout, or by expressing a dominant-negative NOX transgene targeting both enzymes—paradoxically aggravated infarct size, despite overall lower ROS levels. This exacerbated injury was accompanied by severe mitochondrial damage, reduced Hif1α signaling, and enhanced PPARα activity, suggesting that while excessive ROS promote injury, a minimal ROS threshold may be required to sustain mitochondrial integrity and to activate protective transcriptional programs. Thus, suppressing NOX activity below this threshold may become maladaptive, impairing recovery after ischemic stress. Similar context dependence has been observed in vitro. Selective knockdown of NOX4 using siRNA or shRNA improved cardiomyocyte survival and reduced oxidative stress under hypoxia/reoxygenation [81,112] (Table 4). In contrast, simultaneous silencing of NOX4 and NOX2 with either dominant-negative constructs or co-delivery of shRNAs impaired mitochondrial biogenesis and reduced cell viability [81] (Table 2). Interestingly, the same team also reported that cardiac-specific overexpression of NOX4 in mice subjected to I/R had no impact on infarct size or myocardial apoptosis, despite increased O_2_• production compared with non-transgenic controls [81]. This finding suggests that ROS generated by both NOX2 and NOX4 may act cooperatively to amplify oxidative stress and promote myocardial injury. Together, these data provide mechanistic explanations for the apparently contradictory findings outlined in Section 4, highlighting the delicate and crucial balance between beneficial and deleterious ROS signaling in the ischemic heart.

Further insights highlight the difference between transient and permanent NOX4 inhibition. In ex vivo Langendorff-perfused hearts subjected to short I/R protocols (20–25 min ischemia/1 h reperfusion), acute silencing of NOX4 by siRNA reduced infarct size and mitochondrial ROS production [111] (Table 4), whereas permanent inhibition of NOX4—via knockout or dominant-negative constructs—led to increased infarct size, enhanced ER stress, and impaired contractile recovery [77,80] (Table 2). These discrepancies likely reflect the different physiological consequences of transient versus permanent NOX4 inhibition: while acute silencing may attenuate pathological ROS without altering cellular homeostasis, chronic or constitutive suppression may disrupt redox-sensitive protective pathways and provoke compensatory stress responses.

**Figure 3:**
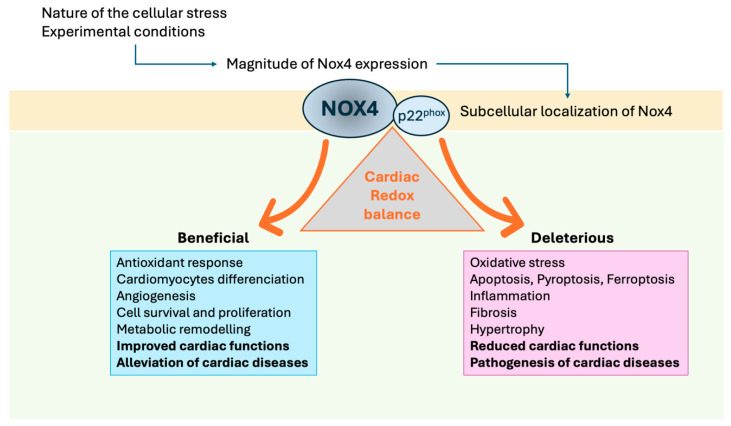
NOX4 functions as a context-dependent modulator of redox signaling, with both protective and pathological roles in the heart.

Taken together, these examples illustrate the highly context-dependent nature of NOX4 activity. Several factors may reconcile these discrepancies. First, NOX4 primarily produces H_2_O_2_, a relatively stable signaling ROS that can trigger protective pathways involved in cell survival, metabolic adaptation, mitochondrial function, and cardiac regeneration. However, the nature and the magnitude of NOX4-induced oxidative stress are not consistently measured across studies, making direct comparisons challenging. Second, the subcellular localization of NOX4 (e.g., in mitochondria, ER or the nucleus) allows for site-specific ROS signaling that may limit widespread oxidative damage and influence whether NOX4 exerts beneficial or deleterious effects. Third, and most importantly, the impact of NOX4 on cardiac health depends not only on its expression level, but also on the nature of the stress, the duration of activation, and the balance between protective signaling and oxidative injury (Figure 3).

Variations in experimental design—including mouse strain, promoter specificity, age and sex of animals, as well as disease model severity and duration—further complicate interpretation. Importantly, the interplay between NOX4 and other NOX isoforms, such as NOX2, adds another layer of complexity, as cooperative or compensatory interactions can dramatically alter redox outcomes. In addition, the presence of multiple NOX4 splice variants (see Section 2.2), with differing capacities to generate ROS and modulate stress responses, as well as the specificity and reliability of experimental tools such as antibodies, inhibitors, and disease-inducing agents [134], may also underlie these discrepancies.

Thus, NOX4 functions as a context-dependent modulator of redox signaling, with both protective and pathological roles in the heart. Its dual nature poses a major challenge for therapeutic targeting, as further discussed in Section 6.

## 6. NOX4: A Potential Therapeutic Target for Heart Disease?

Building on the mechanistic evidence presented in Section 4, where the causal role of NOX4 in cardiac disease was established using transgenic and knockout models, this chapter examines NOX4 as a therapeutic target. We review both direct approaches such as siRNA/shRNA-based silencing or antisense oligonucleotides, and indirect strategies including pharmacological inhibition, angiotensin II receptor blockers (ARBs), and antioxidant treatments. These interventions have been tested in various preclinical models of cardiac pathology, providing proof of concept that modulation of NOX4 can attenuate oxidative stress, inflammation, fibrosis and dysfunction. However, the dual nature of NOX4—exerting both deleterious and adaptive effects depending on context—represents a major challenge for therapeutic translation.

### 6.1. Direct Targeting in Preclinical Models

Given the critical role of NOX4 in promoting cardiac fibrosis and other forms of cardiac dysfunction, targeting NOX4 may offer therapeutic potential for heart diseases. Most direct targeting strategies to date have relied on NOX4 silencing using siRNA or shRNA approaches, in mouse models or cultured cells (Table 4).

#### 6.1.1. Direct Targeting in Preclinical Models of Heart Failure

In vivo, delivery of anti-NOX4-siRNA encapsulated in heart-targeting small extracellular vesicles improved LV contractile function and reduced cardiac hypertrophy in C57BL/6 mice chronically infused with angiotensin II [106] (Table 4). Likewise, our team demonstrated that in Angptl2-knockdown mouse model exhibiting mild aortic valve stenosis and LV systolic dysfunction, cardiac-specific deletion of NOX4 using AAV9-sh-NOX4 attenuated LV contractile dysfunction and increased cardiac antioxidant response [105] (Table 4). In H9c2 cell line stimulated with either isoproterenol, nilotinib, ethanol or doxorubicin, treatment with anti-NOX4-siRNA decreased cell death [89,99,100,102] (Table 4). Similarly, down-regulation of NOX4 using siRNA in human adult cardiomyocytes reduced TNF-α-induced ROS production and upregulation of inflammatory cytokines [16]. Neonatal rat cardiomyocytes stimulated with isoproterenol, angiotensin II and phenylephrine also showed reduced cell size and hypertrophic markers expression after NOX4 knockdown (using siRNA or shRNA) [60,88,103] (Table 4). Finally, silencing NOX4 with siRNA in cardiac fibroblasts significantly reduced NOX4 mRNA levels by over 80%, inhibited O_2_^•^ production in response to TGF-β1, and reduced the TGF-β1-induced expression of smooth muscle alpha-actin, which is involved in the conversion of fibroblasts to myofibroblasts [117]. Together, these findings indicate that NOX4 silencing can blunt pathological hypertrophy and fibrosis in models of heart failure.

However, it is important to note that NOX4 downregulation does not uniformly result in beneficial outcomes. In vitro, knockdown of NOX4 in H9c2 cells stimulated with cobalt dichloride or tunicamycin decreased angiogenic markers expression [28] and cell survival [77], respectively. Similarly, shRNA-mediated knockdown of NOX4 in neonatal rat cardiomyocytes treated with phenylephrine prevented the upregulation of Nrf2 and its downstream cytoprotective genes [76] (Table 2).

#### 6.1.2. Direct Targeting in Preclinical Models of Myocardial Infarction

In mice subjected to LAD coronary artery ligation, administration of in vivo grade anti-NOX4 siRNA abrogated LV contractile dysfunction and attenuated myocardial infarct size and oxidative stress [107] (Table 4). Consistently, silencing NOX4 in the paraventricular nucleus of the hypothalamus attenuated post-MI cardiac dysfunction and peri-infarct apoptosis [135]. The study suggested that the functional benefit observed was primarily due to diminished O_2_^•^ signaling, rather than enhanced H_2_O_2_ levels [135]. In rat isolated beating left atria under hypoxia, specific inhibition of NOX4 using GLX351322 decreased both ROS production and atrial natriuretic peptide (ANP) secretion [108], or reduced ANP secretion alone [109] (Table 4). Collectively, these data suggest that therapeutic targeting of NOX4 may provide benefit in the context of ischemic stress.

In contrast, other studies suggest that NOX4 downregulation may compromise adaptive or protective responses. Knockdown of NOX4 using siRNA in H9c2 cardiomyoblasts after treatment with cobalt chloride, a chemical hypoxia mimetic, decreased redox-regulated Hif1α expression [28] (Table 2). In neonatal rat cardiomyocytes subjected to high glucose, anti-NOX4 shRNA reduced autophagy activation and cell survival [25]. Similarly, anti-NOX4 shRNA in neonatal cardiomyocytes exposed to serum deprivation increased cell necrosis, mitochondrial depolarization and calcium levels [24] (Table 2).

#### 6.1.3. Direct Targeting in Preclinical Models of Ischemia/Reperfusion Injury

In Langendorff-perfused hearts subjected to I/R, treatment with in vivo grade anti-NOX4 siRNA decreased infarct size and mitochondrial superoxide production [111] (Table 4). Similarly, in neonatal rat cardiomyocytes exposed to hypoxia/reoxygenation, downregulation of NOX4 increased cell viability [81,112] (Table 4), supporting a detrimental role for NOX4 during reperfusion injury that can be alleviated by gene silencing. However, this interpretation is tempered by findings that simultaneous knockdown of NOX4 and NOX2 in neonatal rat cardiomyocytes under hypoxia-reoxygenation decreased cell viability and impaired mitochondrial biogenesis [81] (Table 2), highlighting the complex interplay between these NOX isoforms.

#### 6.1.4. Direct Targeting in Preclinical Models of Atrial Fibrillation and Arrhythmia

Targeting NOX4 has shown promise in arrhythmic conditions. In neonatal rat atrial myocytes, siRNA-mediated NOX4 downregulation reduced angiotensin II-induced expression of Kv1.5, a potassium channel selectively involved in atrial repolarization [113] (Table 4). Si-RNAs for NOX2 and NOX4 also attenuated the effects of tachypacing in cultured atrial-derived myocytes (HL-1 cells) by reducing oxidative stress and downregulation of NOX2 and NOX4 [114]. In turn, TGF-β signaling pathway responsible for myofibril degradation was deactivated [114]. Inhibition of NOX4 could therefore mitigate the pathogenesis of AF, suggesting that NOX4 targeting could offer a potential strategy for controlling the progression of this arrhythmia [114] (Table 4).

#### 6.1.5. Direct Targeting in Preclinical Models of Diabetic Cardiomyopathy

In a rat model of diabetic cardiomyopathy using streptozotocin-induced diabetes, treatment with antisense oligonucleotides targeting NOX4 attenuated NOX activity, NOX4 protein expression and ROS generation in the left ventricle, and improved cardiac systolic dysfunction [15]. Additionally, the expression of molecular markers of hypertrophy and myofibrosis, such as fibronectin, collagen, α-SMA, and β-MHC, were also decreased. This suggests that NOX4-derived ROS contribute significantly to cardiomyopathy in the early stages of type 1 diabetes, positioning NOX4 as a promising therapeutic target in this context [15].

Taken together, direct targeting of NOX4 has shown promises in attenuating oxidative stress, inflammation, fibrosis and dysfunction across preclinical models of heart failure, myocardial infarction, I/R injury, AF and diabetic cardiomyopathy (Table 4). However, it should be kept in mind that similar approaches have also yielded opposite outcomes, where NOX4 inhibition exacerbated cardiac dysfunction, impaired myocyte viability and blunted stress-adaptive survival pathways (Table 2).

### 6.2. Indirect Targeting in Preclinical Models

In addition to gene-silencing strategies, NOX4 has been targeted indirectly through small-molecule inhibitors, ARBs and antioxidants (Table 5). These approaches, although often non-specific, provide proof of concept for pharmacological modulation.

#### 6.2.1. Indirect Targeting in Preclinical Models of Heart Failure

Setanaxib (GKT137831), a dual NOX1/NOX4 inhibitor, attenuated cardiac hypertrophy, fibrosis, inflammation and oxidative stress in TAC- [136], doxorubicin- [126], and isoproterenol- [98] induced heart failure models (Table 5). Similarly, Setanaxib reduced cardiomyocytes hypertrophy and ROS production [50,138], decreased inflammasome [98,136] and apoptosis [50] (Table 5). In a cellular model of angiotensin II-induced cardiac fibrosis, Setanaxib decreased both cell proliferation and migration alongside a reduced H_2_O_2_ production [122]. In transgenic mice with mitochondrial-specific NOX4 overexpression, Setanaxib also reduced mitochondrial oxidative stress, decreased LV fibrosis and prevented the development of diastolic dysfunction [85] (Table 5). Apocynin and diphenylene iodonium (DPI), both non-specific NOX inhibitors, likewise reduced cardiac hypertrophy, fibrosis, inflammation and oxidative stress in angiotensin II- [137] and isoproterenol- [139] induced mouse models of heart failure (Table 5). In vitro, DPI also reversed oxidative stress, inflammation and fibrosis in doxorubicin-treated cells [89] (Table 5).

#### 6.2.2. Indirect Targeting in Preclinical Models of Myocardial Infarction and I/R Injury

In C57BL/6 mice subjected to I/R, treatment with Setanaxib attenuated infarct size and LV contractile dysfunction [140] (Table 5). Apocynin also reduced I/R injury and improved contractile recovery in vivo [142] (Table 5). In both Langendorff-perfused heart and mouse cardiomyocytes in culture submitted to hypoxia-reoxygenation, treatment with GLX481304 (a dual inhibitor of NOX2 and NOX4) improved whole heart and cardiac cell contractility, respectively [141] (Table 5).

#### 6.2.3. Indirect Targeting in Preclinical Models of Atrial Fibrillation

Similarly, in preclinical models of AF, apocynin and DPI decreased atrial oxidative stress, reduced Ca^2+^ leaks, and limited atrial remodeling and fibrosis [143,144] (Table 5). In C57BL/6 mice treated with ibrutinib and stimulated by burst pacing, apocynin also reduced AF susceptibility [144] (Table 5). These results are consistent with siRNA studies, reinforcing NOX4 as a promising therapeutic target in AF.

#### 6.2.4. Indirect Targeting in Preclinical Models of Diabetic Cardiomyopathy and Metabolic Disorders

Another indirect strategy to inhibit NOX4 involves the use of ARBs, that reduce NOX4 activation by blocking angiotensin II signaling pathways; these promote oxidative stress and upregulate NOX enzymes including NOX4 in cells [122]. As angiotensin II is a major driver of oxidative stress in the heart, ARBs treatment can lower NOX4-mediated ROS production, thereby attenuating cardiac fibrosis, hypertrophy, and dysfunction associated with pathological remodeling. For example, losartan (a type-1 ARB) treatment in streptozotocin-induced diabetic rats improved myocardial function and suppressed both cardiac and renal fibrosis [146].

Although non-specific, a rational strategy to broadly counteract NOX4- and ROS-mediated cardiac dysfunctions involves the use of antioxidants. In diabetic Akita mice, hyperglycemia and hyperhomocysteinemia led to increased cardiac expression of NOX4, contributing to cardiac remodeling; treatment with the antioxidant tempol reduced NOX4 expression and reversed the remodeling [13]. Similarly, in apolipoprotein E knockout (ApoE-KO) mice, treatment with resveratrol resulted in a downregulation of NOX2 and NOX4 in the heart, which was associated with a reduction in oxidative stress markers such as superoxide, 3-nitrotyrosine, and malondialdehyde [147]. A study in diabetic db/db mice showed that ezetimibe, a cholesterol absorption inhibitor, significantly decreased aortic superoxide levels and reduced the expression of gp91phox, NOX4, Cu/Zn-SOD, and EC-SOD [148]. Ezetimibe also ameliorated cardiac interstitial fibrosis and coronary arterial thickening, indicating that NOX4 may contribute to cardiovascular complications in diabetes [148]. Similarly, Guo et al. [149] observed that in streptozotocin-induced diabetic rats, the expression of NOX4 was significantly elevated in the myocardium, and treatment with rosiglitazone, a drug commonly used to treat type 2 diabetes, attenuated this increase, alongside improvements in heart function. Lastly, in primary neonatal rat cardiomyocytes exposed to high glucose, sodium hydrosulfide treatment reduced ROS and downregulated the expression of mitochondrial NOX4, along with other apoptotic markers, indicating that mitochondrial NOX4 may contribute to oxidative stress and apoptosis in diabetic heart conditions [150]. Altogether, these findings suggest that NOX4 may play a role in the cardiac complications of diabetes, and that targeting its expression could offer therapeutic benefits.

#### 6.2.5. Indirect Targeting in Preclinical Models of Cardiotoxicity and Myocarditis

In models of cardiotoxicity, olmesartan (a type-1 ARB) was tested in combination with daunorubicin, a chemotherapeutic agent known for its cardiac toxicity [151]. Daunorubicin alone increased NOX4 expression along with other oxidative stress markers, resulting in worsened cardiac function. Olmesartan treatment reversed these changes, reducing the protein levels of NOX4 and improving heart function, further implicating NOX4 in daunorubicin-induced cardiac damage [151]. In a rat model of experimental autoimmune myocarditis, treatment with telmisartan (another type-1 ARB) reduced myocardial NOX4 expression and reversed several markers of cardiac dysfunction including oxidative stress, fibrosis, and inflammation [152]. Consistently, in BALB/c mice and HeLa cells stimulated with Coxsackievirus B3, administration of DPI attenuated myocardial inflammation, fibrosis and necrosis and decreased ROS production and apoptosis, respectively [145] (Table 5).

Taken together, indirect inhibition of NOX4 consistently attenuates oxidative stress, fibrosis, and contractile dysfunction across diverse preclinical models of cardiac diseases. To our knowledge, no direct deleterious effects of Setanaxib, apocynin, or DPI on cardiac function have been reported. However, it is important to note that apocynin and DPI are non-specific inhibitors, and thus may affect other cellular oxidases or pathways, potentially leading to indirect effects that have not been fully characterized. Notably, pharmacological inhibition of NOX4 has been mostly explored as a strategy to prevent or reverse NOX4 deleterious effects on the heart. In contrast, therapeutic approaches aimed at modulating NOX4 activity to increase its potentially beneficial effects are only beginning to emerge.

### 6.3. Inhibition of NOX4 in Clinical Settings

The development of specific NOX inhibitors for clinical application remains an ongoing challenge, despite the promising potential of targeting NOXs in various disease settings. Many of the NOX inhibitors described in the literature have either been incompletely characterized, exhibit numerous off-target pharmacologic effects, or lack desirable drug-like qualities, and thus, significant efforts will be required to develop specific, clinically viable inhibitors [153]. While NOX4 inhibitors have not yet been tested in clinical trials specifically for cardiac diseases, preclinical data support their potential therapeutic benefits in conditions such as myocardial infarction, heart failure, and ischemia–reperfusion injury. The existing clinical trials for other indications such as liver and pulmonary diseases [23], provide a foundation for considering NOX4 inhibition as a therapeutic strategy in cardiovascular diseases.

Nevertheless, while specific NOX inhibitors may hold promises, their efficacy and therapeutic value depend on the context and timing of their use. For example, whole-body NOX4 knockout mice did not show protection from diaphragm abnormalities 72 h post-MI, suggesting that systemic inhibition of NOX4 may not benefit patients in the early stages of post-MI recovery [154]. This finding highlights the need for a more nuanced approach to targeting NOX4, with careful consideration of the timing and location of inhibition. A more targeted approach to NOX4 inhibition has shown promise in preclinical models of cardiac hypertrophy: a strategy using cell-derived vesicles (CEVs) loaded with small interfering RNA targeting NOX4 (siNOX4) was developed [106]. This approach enhanced the anti-hypertrophic effects of the treatment by ensuring heart-specific delivery of the therapeutic siRNA. When administered intravenously to angiotensin II-treated mice, the CEVs@siNOX4 treatment significantly improved cardiac function, reduced fibrosis, and decreased the cardiomyocyte cross-sectional area, all with minimal side effects [106]. This targeted delivery system shows promise as an efficient strategy for treating cardiac hypertrophy, underscoring the potential of localized, precise NOX4 inhibition as a therapeutic avenue.

Taken together, the key to success will lie in overcoming the challenges of specificity, timing, and delivery to ensure that clinical therapeutic interventions can harness the beneficial effects of NOX4 inhibition without unwanted side effects.

## 7. Conclusions

NOX4 plays a dual role in heart health and disease. It contributes to oxidative stress, fibrosis, and maladaptive remodeling in conditions where its expression is typically upregulated, like heart failure, myocardial infarction, ischemia/reperfusion (I/R), arrhythmias, and diabetes. Inhibiting or knocking out NOX4 in these contexts has shown therapeutic benefits. However, NOX4 also supports cardiac function by regulating energy metabolism, mitigating oxidative stress as a redox sensor, and promoting remodeling and angiogenesis. These opposing roles underscore its context-dependent effects.

In human heart failure, most studies report increased NOX4 expression in the failing myocardium, though causality remains unclear. Elevated NOX4 levels have been associated with markers of mitochondrial oxidative stress, fibrosis, pyroptosis, and immune activation, suggesting a possible deleterious role. Yet other findings indicate that NOX4 upregulation may instead reflect a compensatory antioxidant response that becomes insufficient under chronic stress. Contradictory evidence, including reports of unchanged or even reduced NOX4 levels in specific heart failure subtypes, further underscores the complexity of its role. This uncertainty is largely due to the inherent limitations and challenges of working with human tissue samples. Collectively, these findings highlight the need for more mechanistic studies to determine whether NOX4 is a driver of cardiac pathology or a marker of an adaptive response to stress in human heart failure.

As a therapeutic target, NOX4 offers both promises and complexities. The challenge lies in modulating its activity to preserve protective effects, while minimizing harm. Although developing specific and effective NOX4 inhibitors is difficult, novel strategies like targeted siRNA delivery via cell-derived vesicles hold potential. Moving forward, balancing NOX4’s harmful and beneficial functions will be key to successful heart disease therapies.

The answer to the question “Is NOX4 induction associated with increased oxidative stress or increased antioxidant response?” is likely context-dependent, as NOX4 may be linked to either outcome depending on the specific cellular environment and pathological conditions. In some contexts, NOX4 is viewed as a sensor of antioxidant activity [155] and part of an adaptive stress response to physiological insults [32,33,71]. In contrast, other studies identify NOX4 as a major contributor to the detrimental effects of chronic oxidative stress on cardiac function [4,60,81,85]. Collectively, the findings suggest that NOX4 may act as a redox sensor, initiating signaling pathways to restore oxidative homeostasis when the cellular antioxidant capacity remains sufficient and the oxidative stress is not yet overwhelming. Ultimately, the role of cardiac NOX4, whether protective or pathogenic, is highly context-dependent and should not be interpreted through a singular or uniform perspective.

Although the similarity in the names NOX (the enzyme) and Nox (the primordial goddess of the night) is purely coincidental, there are some intriguing metaphorical parallels that can be drawn. Like the goddess Nox who embodies both creation and destruction, NOX4 plays a crucial role in various biological processes, yet its activity can also lead to oxidative stress and cellular damage, contributing to cardiovascular diseases.

## 8. Future Perspectives on NOX4 and Cardiac Function

Since NOX4 plays a dual role in the heart, acting as both a mediator of oxidative damage and a regulator of protective signaling depending on context, levels of expression, and subcellular localization, this nuanced role positions NOX4 not simply as a pathological target, but as a potential modulator of redox homeostasis. Due to its involvement in cardiac fibrosis, hypertrophy, apoptosis, and mitochondrial dysfunction, targeting NOX4 pharmacologically is promising in conditions like heart failure, I/R injury, and cardiomyopathies. However, because low levels of NOX4-derived ROS are also crucial for adaptive signaling (e.g., via Nrf2 or Hif1α), therapeutic inhibition needs to be finely tuned to avoid impairing protective mechanisms. For example, NOX4 is upregulated with age and may contribute to age-associated cardiac dysfunction. However, its role in stem cell differentiation and cardiac development suggests that it could also be harnessed for regenerative strategies, particularly in tissue engineering or post-injury repair. NOX4 is therefore a double-edged therapeutic target. The existence of NOX4 splice variants with distinct localizations and functions adds a layer of complexity. Future therapies may aim to selectively modulate specific isoforms to maximize benefit while minimizing harm.

Future directions may involve the development of isoform- or localization-specific NOX4 inhibitors, targeted delivery systems (e.g., siRNA via nanoparticles or vesicles), better tools to monitor NOX4 activity in vivo, and clinical trials to evaluate NOX4 modulation in human cardiac diseases.

## Figures and Tables

**Table 1:** Beneficial roles of induced NOX4 expression in cardiac homeostasis and pathology.

Model	Stimuli	Main Outcomes
**Heart homeostasis—resistance to stress, cardiac metabolism**		
Neonatal rat cardiomyocytes	NOX4 overexpression (adenovirus)	Increased cell proliferation, cell cycle pathway activation (c-myc, cyclin D2) and fatty acid oxidation via protein O-GlcNAcylation [29,69]
Tg NOX4 mice; cardiac specific—αMHC promoter (C57BL/6 background)		Increased cardiac antioxidant/detoxifying Nrf2-regulated genes expression, cardiomyocytes cell cycling activity (cyclin D2), proliferative capacity and fatty acid oxidation via protein O-GlcNAcylation [29,69,71]
Tg NOX4 mice; endothelium specific—tie2 promoter (C57BL/6 background)		Eccentric cardiac hypertrophy with a physiological pattern of slightly enhanced LV systolic function [73]
**Heart failure**		
Tg NOX4 mice; cardiac specific—αMHC promoter (C57BL/6 background)	TAC (suprarenal banding); 9 weeks	Preserved systolic/diastolic function and decreased cardiac hypertrophy and fibrosis; Increased angiogenic markers expression (VEGF, Hif1α); Increased fatty acid oxidation via protein O-GlcNAcylation [28,29]
Tg NOX4 mice; endothelium specific—tie2 promoter (C57BL/6 background)	Angiotensin II	Protection against myocardial fibrosis, inflammatory cell infiltration and endothelial activation [73]
**Myocardial ischemia**		
Neonatal rat cardiomyocytes; Rat H9c2 cardiomyoblasts	Hypoxia + NOX4 overexpression (adenovirus)	Increased angiogenic marker expressions (VEGF, Hif1α) [28]
Tg NOX4 mice; cardiac specific—αMHC promoter (C57BL/6 background)	Left coronary artery ligation	Increased survival rate, preserved LV systolic and diastolic function; Decreased cardiac hypertrophy, fibrosis and MMP2 activity [74]
**Myocardial ischemia/reperfusion injury**		
Tg NOX4 mice; cardiac specific—αMHC promoter (C57BL/6 background)	I (30 min)/R (3–7 days)	Preserved LV systolic function; Increased macrophages proportion and polarization toward an M2 phenotype [74]

αMHC: alpha-myosin heavy chain; Hif1α: hypoxia-inducible factor-1 alpha; I/R: ischemia/reperfusion; LV: left ventricular; MMP2: matrix metalloproteinase 2; Nrf2: nuclear factor erythroid 2–related factor 2; TAC: transverse aortic constriction; Tg: transgenic; VEGF: vascular endothelial growth factor.

**Table 2:** Direct decrease in cardiac NOX4 leads to deleterious effects in animal and cellular models of cardiac diseases.

Compound/Method	Specie/Cell Type	Stimuli	Main Outcomes
**Heart differentiation and resistance to stress**			
Tg inducible cardiomyocytes-specific NOX4-KO mice; (C57BL/6 background)		Acute exercise stress	Decreased LV contractile function, cardiac Nrf2 and antioxidant markers expression [72]
riNOX4 (ribozyme); anti-NOX4 siRNA or shRNA	Murine embryonic stem cells	Differentiation protocol	Decreased beating activity and cardiac genes expression (Nkx2.5, MEF2C) [64,66]
		Differentiation protocol + thalidomide	Decreased cardiac (α-actinin-positive) area formation and impaired cardiomyogenesis [68]
		Differentiation protocol + static magnetic fields	Decreased cardiac foci area and mRNA expression of cardiac genes (MLC2, α- and β-MHC, Nkx-2.5, GATA-4) [65]
	Murine embryonal carcinoma cells	Differentiation protocol	Decreased expression of cardiac markers (GATA-4, MEF2C, α-MHC) [67]
	Neonatal rat cardiomyocytes		Decreased protein O-GlcNAcylation and fatty acid oxidation [29]
**Heart failure**			
NOX4-KO mice; systemic (C57BL/6 background)		TAC (aortic arch); 2 weeks	Exaggerated cardiac dysfunction and increased cardiac hypertrophy and fibrosis; Decreased angiogenic marker expressions (VEGF, Hif1α) [75]
		TAC (suprarenal aortic banding); 6 weeks	Exaggerated cardiac dysfunction and increased cardiac hypertrophy and fibrosis; Decreased angiogenic marker expressions (VEGF, Hif1α) [28]
			Decreased cardiac Nrf2 expression and its target cytoprotective genes [76]
NOX4-KO mice; endothelium specific (C57BL/6 background)		TAC (suprarenal aortic banding); 6 weeks	Exaggerated cardiac dysfunction and increased cardiac hypertrophy and fibrosis; Decreased angiogenic marker expressions [75]
Tg inducible cardiomyocytes-specific NOX4-KO mice; (C57BL/6 background)			
Anti-NOX4 shRNA	H9c2 cells	Tunicamycin	Decreased myocyte survival and increased cell apoptosis [77]
	Neonatal rat cardiomyo-cytes	Phenylephrine	Decreased cardiac Nrf2 expression and its target cytoprotective genes [76]
NOX4-disrupted H9c2 cells by CRISPR-Cas9 genome editing		Acrolein or methyl vinyl ketone (MVK)	Exacerbated the decrease in cell viability [78]
Cardiomyocytes isolated from NOX4-KO mice (Jackson Lab; #022996)		Isoproterenol + insulin	Prevented insulin-induced attenuation of cardiomyocyte contractility and β-adrenergic signaling activity [79]
**Myocardial ischemia**			
NOX4-KO mice; cardiac specific (C57BL/6J background)		Fasting (48 h)	Decreased LV contractile function, myocardial ATP content and autophagy activation [25]
		Prolonged ischemia (3 h)	Increased infarct size and necrosis [25]
Anti-NOX4 siRNA or shRNA	Neonatal rat cardiomyocytes	Glucose deprivation	Decreased ROS production, autophagy activation and cell survival; Increased cell apoptosis [25]
		Serum deprivation	Increased cell death (necrosis), mitochondrial depolarization and calcium levels [24]
	H9c2 cells	Cobalt chloride (CoCl_2_)	Decreased redox-regulated Hif1α expression [28]
**Myocardial ischemia/reperfusion injury**			
NOX4-KO mice; systemic (C57BL/6 background)		I (25 min)/R (1 h) in Langendorff-perfused heart	Increased infarct size and ER stress-associated cell death [77]
		I (25 min)/R (30 min) in Langendorff-perfused heart	Higher cardiomyocyte death (release of cTnI); Lower recovery of contractile function after reperfusion [24]
Tg-P437H (a dominant negative of NOX4; competes with both NOX4 and Nox2) mice; cardiac specific (C57BL/6J background)		I (25 min)/R (1 h) in Langendorff-perfused heart	Increased infarct size and mitochondrial ROS production; Decreased NAD(P)^+^/NAD(P)H, myocardial energetics and contractile performance [80]
		I (30 min)/R (24 h)	Increased infarct size and myocardial apoptosis and triglyceride content; Decreased cardiac ROS production and angiogenic marker (Hif1α) expression [81]
NOX4-KO; cardiac specific × Nox2-KO; systemic mice (C57BL/6J background)		I (30 min)/R (24 h)	Increased infarct size and myocardial apoptosis and triglyceride content; Decreased cardiac ROS production and angiogenic marker (Hif1α) expression [81]
Dominant-negative Nox or shNOX4 + shNox2	Neonatal rat cardiomyocytes	Hypoxia (12 h)/reoxygenation (24 h)	Decreased cell viability, mitochondrial biogenesis and O_2_^−^ production [81]

αMHC: alpha-myosin heavy chain; bMHC: beta-myosin heavy chain; cTnI: cardiac troponin I; ER: endoplasmic reticulum; Hif1α: hypoxia-inducible factor-1 alpha; I/R: ischemia/reperfusion; KO: knockout; LV: left ventricular; MLC2: myosin light chain 2; MEF2C: myocyte enhancer factor 2C; Nkx2.5: NK2 homeobox 5; Nrf2: nuclear factor erythroid 2–related factor 2; TAC: transverse aortic constriction; Tg: transgenic; VEGF: vascular endothelial growth factor.

**Table 3:** Deleterious roles of induced NOX4 expression in cardiac pathology.

Model	Stimuli	Main Outcomes
**Heart failure**		
Tg NOX4 mouse; cardiac specific (FVB background)	TAC (aortic arch); 2 weeks	Exacerbated LV contractile dysfunction, cardiac hypertrophy and apoptosis [4,49]
Tg NOX4 mouse; cardiac specific (FVB background)	Continuous infusion of phenylephrine	Exacerbated cardiac hypertrophy and oxidative stress [60]
Tg NOX4 mouse; mitochondrial-specific (NOX4TG61; C57BL/6J background)		Diastolic dysfunction, increased myocardial fibrosis and Ca^2+^ influx, altered/disrupted Z-disc structure; Mitochondrial oxidative stress, DNA damage and dysfunction [85]
H9c2 cells	NOX4 overexpression	Increased cell death [86] and cytokine production [87]
Neonatal rat cardiomyocytes		Increased cell apoptosis [63], cell size and hypertrophic markers expression [88]
H9c2 cells	Doxorubicin + NOX4 overexpression	Aggravated doxorubicin-induced oxidative stress and apoptosis [89]
AC16 cells	Doxorubicin + HucMSC-EVs + NOX4 overexpression	Abolished the protective effects of HucMSC-EVs on cell apoptosis and oxidative stress [90]
Neonatal rat cardiomyocytes	Isoproterenol + FGF18 and NOX4 overexpression	Abolished the protective effect of FGF18 and aggravates cardiomyocyte hypertrophy, apoptosis and fibrosis [50]
C57BL/6J mouse	Isoproterenol + AAV9-NOX4 and AAV9-Prx-3	Abrogated the improvement of LV contractile dysfunction and mitigates the anti-fibrosis effects of Prx-3 [91]
**Myocardial infarction (MI)**		
Sprague-Dawley rat	LAD coronary artery ligation + NOX4 and Endostatin adenoviral overexpression	Abrogated endostatin-mediated beneficial effects on MI: increased LV systolic dysfunction, myocardial oxidative stress and fibrosis [92]
	LAD coronary artery ligation + Tanshinone IIA + NOX4 adenoviral overexpression	Abrogated Tanshinone IIA-mediated beneficial effects on MI: increased LV systolic dysfunction and myocardial fibrosis [93]
**Myocardial ischemia/reperfusion injury (I/R)**		
Tg NOX4 mouse; cardiac specific (FVB background)	I (25 min)/R (1 h) in a Langendorff system	Increased infarct size and decreased cardiac energetics and contractile performance [80]
**Atrial fibrillation and arrythmia**		
Zebrafish embryo	Human NOX4 RNA—injection at one-cell stage	Phenotype of irregular heartbeats, with increased superoxide production and CaMKII activation [94]
**Duchenne muscular dystrophy**		
Tg Mdx mice	NOX4 overexpression (AAV9)	Accelerated cardiac remodeling and fibrosis [95]
**Age-related heart failure**		
Tg NOX4 mice; cardiac specific (FVB background)	Aging; 13–14 months	LV contractile dysfunction without cardiac hypertrophy; increased myocardial fibrosis, apoptosis, oxidative stress and mitochondrial dysfunction [63]

CaMKII: calcium/calmodulin-dependent protein kinase II; FGF18: fibroblast growth factor 18; HucMSC-EVs: human mesenchymal stem cell-derived extracellular vesicles; I/R: ischemia/reperfusion; LAD: left anterior descending; LV: left ventricular; MI: myocardial infarction; Prx-3: peroxiredoxin 3; TAC: transverse aortic constriction; Tg: transgenic.

**Table 4:** Targeted reduction in cardiac NOX4 produces beneficial outcomes in preclinical models of heart diseases.

Compound/Method	Specie/Cell Type	Stimuli	Main Outcomes
**Heart failure**			
NOX4-KO mice; cardiac specific (C57BL/6J background)		TAC (aortic arch; 2–4 weeks)	Improved LV contractile function and decreased cardiac hypertrophy, interstitial fibrosis and apoptosis [4,60]
		Phenylephrine—continuous infusion	Decreased cardiac hypertrophy and O_2_• production [60]
		Isoproterenol (ISO)	Preserved diastolic function; Reduced inflammasome activation, cytokine levels, pro-inflammatory macrophage subpopulations, cardiac fibroblasts activation and interstitial fibrosis [98]
NOX4-KO mice; systemic (C57BL/6 background)		Aortocaval fistula (Shunt); 2 weeks	Limited LV dilatation and hypertrophy, without effect on cardiac fibrosis [96]
NOX4-KO mice (cardiac specific) × FYN-KO mice		TAC (aortic arch); 2 weeks	Rescued the exaggerated cardiac hypertrophy; Decreased cardiac ROS production and apoptosis [49]
NOX4-KO (Jackson Lab; #022996) Elmo1-hypermorphic mouse			Alleviated the dilated cardiomyopathy phenotype: restored cardiac morphology and LV contractile function [97]
Anti-NOX4 siRNA	Human adult cardiomyocytes	TNF-α	Decreased ROS production and upregulation of inflammatory cytokines (IL-1β, IL-6) [16]
Anti-NOX4 siRNA	H9c2 cell line (rat embryonic ventricular myocytes)	Isoproterenol (ISO)	Decreased mitochondrial oxidative stress and apoptotic signaling [99]
		Nilotinib	Decreased cell apoptosis, mitochondrial dysfunction and ROS production [100]
		Lunar dust simulant	Decreased collagen1α1 and Nrf2 expression, ROS production [101]
		Ethanol	Decreased cell autophagy and apoptosis [102]
		Doxorubicin	Decreased cell apoptosis and ROS production [89]
Anti-NOX4 siRNA	Neonatal rat cardiomyocytes	Isoproterenol (ISO)	Decreased cell size, hypertrophic markers expression and ROS production [103]
		Angiotensin II	Decreased cell size, hypertrophic markers expression and ROS production [88,103]
Anti-NOX4 shRNA		Phenylephrine	Decreased cell size, ANP expression, ROS production, HDAC4 oxidation and nuclear exit [60]
Anti-NOX4 shRNA	AC16 cells	KLF5 overexpression	Decreased ROS production and increased mitochondrial abundance [104]
Anti-NOX4 shRNA	Tg Angptl2-KD mice; systemic (C57BL/6 background)		Attenuated LV contractile dysfunction and increased cardiac antioxidant response [105]
Heart-targeting small extracellular vesicles + anti-NOX4 siRNA	C57BL/6 mouse	Angiotensin II	Decreased LV contractile dysfunction, cardiac hypertrophy and fibrosis [106]
	iPSC-vCM		Decreased cardiomyocytes size and hypertrophic markers expression [106]
**Myocardial ischemia**			
NOX4-KO mice; systemic (C57BL/6J background)		LAD coronary artery ligation	Decreased infarct area and myocardial oxidative stress, DNA damage, macrophage infiltration and apoptosis [87]
In vivo grade anti-NOX4 siRNA	C57BL/6J mouse	LAD coronary artery ligation	Abrogated LV contractile dysfunction and attenuated myocardial infarct size and oxidative stress [107]
GLX351322	Isolated beating left atria (Sprague-Dawley rat)	Hypoxia	Decreased ROS production and ANP secretion [108]; Decreased ANP secretion [109]
GLX351322	Isolated beating left atria (Sprague-Dawley rat)	Sulfated CCK-8 (CCK-8s)	Decreased ANP secretion and ROS production [110]
**Myocardial ischemia/reperfusion injury**			
NOX4-KO mice; systemic and cardiac-specific (C57BL/6J background)		I (30 min)/R (24 h)	Decreased myocardial infarct size/area at risk, myocardial apoptosis and O_2_• production [81]
In vivo grade anti-NOX4 siRNA	Langendorff- perfused hearts (C57BL6 mouse)	I (20 min)/R (60 min)	Decreased infarct size and mitochondrial superoxide production [111]
Anti-NOX4 siRNA	Neonatal rat cardiomyocytes	Hypoxia (30 min)/reoxygenation (24 h)	Increased cell viability; Decreased inflammatory markers expression and ROS production [112]
Anti-NOX4 shRNA		Hypoxia (12 h)/reoxygenation (24 h)	Increased cell viability and decreased O_2_• production [81]
**Atrial fibrillation and arrythmia**			
Anti-NOX4 siRNA	Neonatal rat atrial myocytes	Angiotensin II	Decreased ROS production and Kv1.5 expression [113]
	HL-1 cell line (atrial myocytes)	Tachypacing	Decreased ROS production and myosin degradation [114]
**Cardiac fibrosis**			
Plumbagin (specific NOX4 inhibitor)	Adult rat atrial fibroblasts	TGF-β1	Decreased ROS production and fibronectin expression [115]
Anti-NOX4 siRNA			
Anti-NOX4 siRNA	Human cardiac fibroblasts (from failing LVs)		Decreased myofibroblast transformation, collagen synthesis and mitochondrial oxidative stress [116]
Anti-NOX4 siRNA	Human cardiac fibroblasts		Inhibited O_2_• production, reduced TGF-β1-induced expression of α-SMA and decreased cardiac fibroblasts differentiation into myofibroblasts [117]
Anti-NOX4 siRNA	Human cardiac fibroblasts	TXNDX5 overexpression	Decreased cell proliferation, ROS production and ECM protein upregulation [118]
Anti-NOX4 siRNA	Neonatal rat cardiac fibroblasts	Angiotensin II	Limited fibrotic response (decreased MMP-2, MMP-9, α-SMA expression) [119]
			Decreased pro-fibrotic marker (CTGF) expression [120]
Anti-NOX4 siRNA	Adult mouse cardiac fibroblasts	Angiotensin II	Decreased cell proliferation, migration and H_2_O_2_ generation [121,122]
		IL-18	Decreased cell migration, H_2_O_2_ generation and MMP9 expression [123]
Anti-NOX4 siRNA	Adult rat ventricular fibroblasts	H_2_O_2_	Decreased cell proliferation and fibronectin levels [124]

ANP: atrial natriuretic peptide; CTGF: connective tissue growth factor; ECM: extracellular matrix; HDAC4: Histone Deacetylase 4; ISO: isoproterenol; iPSC-vCM: induced pluripotent stem cell-derived ventricular cardiomyocytes; I/R: ischemia/reperfusion; KO: knockout; LAD: left anterior descending coronary artery; MMP: matrix metalloproteinase; Nrf2: nuclear factor erythroid 2–related factor 2; TAC: transverse aortic constriction.

**Table 5:** Non-specific Nox inhibitors exhibit beneficial cardiac effects in animal and cellular models of cardiac diseases.

Compound	Specie/Cell Type	Stimuli	Main Outcomes
**Heart failure**			
Setanaxib (GKT137831)	Tg NOX4 mice; mitochondrial-specific (NOX4TG61; C57BL/6J background)		Inhibited mitochondrial oxidative stress, decreased LV fibrosis and prevented development of diastolic dysfunction [85]
Setanaxib (GKT137831)	Sprague-Dawley rat	Abdominal aortic constriction	Attenuated cardiac hypertrophy, fibrosis, oxidative stress and inflammation [136]
Apocynin	C57BL/6 mouse	Angiotensin II	Decreased cardiac hypertrophy, fibrosis and oxidative stress [137]
Setanaxib (GKT137831)	Neonatal rat ventricular myocytes		Attenuated cardiac hypertrophy and ROS production [138]
Setanaxib (GKT137831)	C57BL/6J mouse	Doxorubicin	Attenuated LV contractile dysfunction and myocardial damage, pyroptosis and inflammation [126]
Setanaxib (GKT137831)	H9c2 cells		Reduced ROS production, NLRP3 inflammasome activation and mitochondrial fission [126]
Diphenylene iodonium	H9c2 cells		Decreased cell apoptosis and oxidative stress [89]
Setanaxib (GKT137831)	Neonatal rat cardiomyocytes		Reduced NLRP3 inflammasome activation and pyroptosis [126]
Setanaxib (GKT137831)	Wild-type mouse	Isoproterenol	Preserved diastolic function; Decreased cardiac mitochondrial ROS production, inflammation and fibrosis [98]
Apocynin	AQP4-KO mice		Decreased cardiac hypertrophy, ROS production and inflammation [139]
Setanaxib (GKT137831)	Neonatal rat cardiomyocytes		Decreased cell size and hypertrophy markers expression, fibrosis, apoptosis and ROS production [50]
Setanaxib (GKT137831)	Neonatal mouse cardiomyocytes		Decreased inflammasome activity [98]
**Myocardial ischemia/reperfusion injury**			
Setanaxib (GKT137831)	C57BL/6 mouse	Ischemia–reperfusion	Attenuated infarct size and LV contractile dysfunction [140]
GLX481304	C57BL/6 mouse	Hypoxia- reoxygenation in Langendorff-perfused hearts	Improved whole heart contractility [141]
	Mouse cardiomyocytes	Hypoxia-reoxygenation	Decreased ROS production and improved cell contractility [141]
Apocynin	HAX-1-KO mice	Ischemia–reperfusion in Langendorff-perfused heart	Decreased infarct size and improves contractile recovery [142]
**Atrial fibrillation and arrythmia**			
Diphenylene iodonium	C57BL/6 mouse	TAC (aortic arch); 4 weeks	Decreased left atrium oxidative stress and diastolic sarcoplasmic reticulum Ca^2+^ leak [143]
Apocynin	C57BL/6 mouse		
Apocynin	C57BL/6 mouse	Ibrutinib + burst pacing	Reduced AF susceptibility and atrial electrical remodeling and fibrosis [144]
**Cardiac fibrosis**			
Setanaxib (GKT137831)	Adult mouse cardiac fibroblasts	Angiotensin II	Decreased cell proliferation and migration, H_2_O_2_ generation and AT1/NOX4 binding [122]
	Human primary cardiac fibroblast	TXNDX5 overexpression	Decreased cell proliferation, ROS production and ECM protein upregulation [118]
**Myocarditis**			
Diphenylene iodonium	BALB/c mouse	Coxsackievirus B3	Attenuated myocardial inflammation, fibrosis and necrosis [145]
	HeLa cells		Decreased ROS production and apoptosis [145]

AF: atrial fibrillation; AQP4: aquaporin 4; AT1: angiotensin II type 1 receptors; ECM: extracellular matrix; HAX-1: HS1-associated protein X-1; KO: knockout; LV: left ventricular; NLRP3: NOD-like receptor family pyrin domain containing 3; TAC: transverse aortic constriction; Tg: transgenic; TXNDX5: thioredoxin domain-containing protein 5.
